# A rabbit model of acute bacteremia and sepsis caused by vancomycin-resistant *Enterococcus faecium*

**DOI:** 10.3389/fcimb.2026.1619290

**Published:** 2026-04-16

**Authors:** Karen Flores-Moreno, René Arredondo-Hernández, Claudia Mayoral-Terán, Yolanda López-Vidal

**Affiliations:** 1 Programa de Inmunología Molecular Microbiana, Departamento de Microbiología y Parasitología, Facultad de Medicina, Universidad Nacional Autónoma de México, Ciudad de México, Mexico; 2 Laboratorio de Microbioma, División de Investigación, Facultad de Medicina, Universidad Nacional Autónoma de México, Ciudad de México, Mexico

**Keywords:** bacteremia, histopathology, monoclonal antibody therapy, rabbit model, vancomycin-resistant *Enterococcus faecium* (VREF)

## Abstract

**Background:**

Vancomycin-resistant *Enterococcus faecium* (VREF) is a leading cause of nosocomial bacteremia, with mortality rates exceeding 60%. Current animal models lack translational relevance due to the divergence in immune systems between rodents and humans.

**Objectives:**

To establish a rabbit model of *Enterococcus faecium* infection for evaluating therapeutic regimes, specifically monoclonal antibody (mAb) therapy.

**Methods:**

We established a reproducible rabbit model of acute VREF bacteremia using New Zealand White rabbits (*n* = 20) intravenously challenged with 1 × 10^9^ CFU/g of VREF (strain E155). The animals were divided into three groups: uninfected controls (*n* = 6), infected untreated (*n* = 7), and infected treated with mAb 8AP (*n* = 7). We histopathologically examined the kidneys, lungs, spleen, heart, and liver, and quantified bacterial loads. Plasma cytokine levels were measured to assess inflammatory responses.

**Results:**

Infected untreated rabbits exhibited significant weight loss, sustained fever (>40 °C, peaking at 39h), and 20% mortality within 12h. Systemic bacterial dissemination was confirmed in all organs examined, with the spleen showing the highest bacterial load and the liver exhibiting the most severe histopathological damage (necrosis and suppurative inflammation). The mAb 8AP treatment significantly improved clinical outcomes: heart and respiratory rates decreased, and body weight increased compared with untreated infected rabbits. Cytokine profiling showed elevated levels of IL-6, IL-8, and IL-10, consistent with systemic inflammatory response syndrome (SIRS), with the mAb treatment showing a trend toward immune modulation.

**Conclusions:**

This model successfully simulates human VREF bacteremia and demonstrates the therapeutic potential of mAb 8AP. The rabbit model provides a clinically relevant platform for evaluating novel immunotherapies for VREF infections.

## 1 Introduction

*Enterococcus faecium* has an extraordinarily malleable genome, with a large number of mobile genetic elements (MGE) encoding antimicrobial resistance genes (Arias and Murray, 2012; Gilmore et al., 2014; Wei et al., 2024). At present, *E. faecium* causes significant numbers of healthcare-associated infections, with high mortality rates (>60%), particularly among vancomycin-resistant cases (Munita et al., 2012; Guzman Prieto et al., 2016). Unlike *Enterococcus faecalis*, *E. faecium* is associated with higher intrahospital mortality. The World Health Organization has designated vancomycin-resistant *E. faecium* (VREF) as a high-priority pathogen requiring novel therapeutic approaches (Guzman Prieto et al., 2016; Yan et al., 2017; Raven et al., 2018; WHO, 2024).

Current animal models for studying VREF infections have significant limitations. Murine models, while widely used, fail to adequately mimic human immune dynamics due to differences in Toll-like receptor (TLR) signaling pathways and substantial genetic divergence from humans (Heinz et al., 2003; Vaure and Liu, 2014; Goh et al., 2017). Rabbit models address these limitations through three key advantages: (1) phylogenetic proximity to primates with less genetic diversity than in mice (Soares et al., 2022), (2) intermediate body size enabling serial sampling and detailed monitoring (Goh et al., 2017), and (3) TLR distribution and immune kinetics that closely mirror human responses (Vaure and Liu, 2014). These attributes enable rabbits to more accurately replicate human pathophysiology, particularly in conditions characterized by high mortality and severe organ damage.

The rabbit model can address critical gaps in understanding VREF pathogenesis, particularly factors associated with early mortality such as hepatic dysfunction, that remain poorly characterized in bacteremia despite its central role in sepsis pathophysiology (Bosze and H. L. M., 2006). Whereas local damage and cross-organ communication events, including kidney, spleen, and cardiac disease, preclude direct analysis of organ-specific damage caused by *E. faecium* bacteremia, both require further investigation (Heinz et al., 2003; Bosze and H. L. M., 2006; Zanotti-Cavazzoni and Goldfarb, 2009; Salgado-Pabón and Schlievert, 2014; Peng and K. J. H. K., 2015; Goh et al., 2017). The escalating burden of VREF, with >80% of enterococcal isolates in some regions showing vancomycin resistance, necessitates the development of translational models that bridge the gap between preclinical and clinical research (Weiner et al., 2016).

Therefore, the objective of this study was to establish a rabbit model of acute VREF bacteremia to evaluate therapeutic regimens, particularly novel immunotherapeutics such as monoclonal antibodies. This model provides a platform for testing interventions under conditions that mirror human bacteremia, including immune evasion mechanisms and antibiotic failure trajectories (Sauvat et al., 2024).

## 2 Materials and methods

### 2.1 Bacterial strains and culture conditions

*Enterococcus faecium* strain E155, a vancomycin-resistant clinical isolate (MIC >256 μg/ml) from a nosocomial outbreak, was used in this study. The strain was confirmed as *E. faecium* by 16S rRNA gene sequencing, and vancomycin resistance was verified by the E-test. The strain was stored at −80 °C in brain heart infusion (BHI) broth containing 20% glycerol.

For experimental infections, the bacteria were cultured in trypticase soy (TS) broth at 37 °C with shaking (180 rpm) for 16–18h to mid-log phase (OD_600_ = 0.6–0.8). Bacterial cells were harvested by centrifugation (4,000 × g, 10 min, 4 °C), washed three times with sterile phosphate-buffered saline (PBS, pH 7.4), and resuspended in PBS to a concentration of 1 × 10^9^ CFU/ml. The inoculum concentration and bacterial species were confirmed by plating serial dilutions on TS agar and counting colonies after 24h of incubation at 37 °C. The bacterial species was confirmed by plating inoculum in CHROMO agar VRE.

### 2.2 Growth curve and bacterial characterization

A growth curve was generated by inoculating 100 ml of TS broth with an overnight culture (1:100 dilution) and monitoring the optical density at 600 nm hourly for 24h. Samples were collected at each timepoint for CFU enumeration. The growth curve was used to determine the optimal harvest time for infection studies (i.e., the mid-log phase).

Bacterial protein profiles were analyzed by SDS-PAGE. Bacterial cells were lysed by sonication, and protein concentration was determined by Bradford assay. Proteins (20 μg per lane) were separated on 12% polyacrylamide gels and visualized by Coomassie blue staining.

### 2.3 Monoclonal antibody production

Monoclonal antibodies against VREF E155 were generated using standard hybridoma technology. BALB/c mice (6–8 weeks old, *n* = 5) were immunized with 50 µg of either VREF membrane or cell wall proteins. Each mouse received six subcutaneous injections in the left leg. Blood samples were collected before each immunization to monitor antibody titers against the VREF membrane or cell-wall components. P3-X63-Ag8 myeloma cells (7.1 × 10^5^ cells) using polyethylene glycol (PEG 1500) to generate hybridomas.

Hybridoma supernatants were screened by ELISA against the VREF E155 cell wall and membrane fractions. Positive clones were subcloned by limiting dilution and expanded for antibody production. Antibodies were purified from the culture supernatants using Protein G affinity chromatography and concentrated using Amicon Ultra centrifugal filters (30 kDa MWCO, Millipore) to 75% (v/v). Antibody concentration was determined by the Bradford assay, and purity was confirmed by SDS-PAGE. Isotype determination was performed using a commercial kit (Sigma-Aldrich).

### 2.4 Opsonophagocytic assay

An opsonophagocytic killing assay was performed to evaluate the ability of monoclonal antibodies to enhance bacterial killing by polymorphonuclear cells (PMNs). Human PMNs were isolated from fresh heparinized blood by density gradient centrifugation using Ficoll-Paque medium. PMNs (1 × 10^6^ cells) were incubated with VREF E155 (1 × 10^5^ CFU) and monoclonal antibodies (10 μg/ml) in the presence of 10% baby rabbit complement (Cedarlane Laboratories) for 90 min at 37 °C with gentle agitation.

The samples were serially diluted and plated on TS and CHROMO agar VRE to enumerate surviving bacteria. Percent killing was calculated as: [(CFU without antibody − CFU with antibody)/CFU without antibody] × 100. Each assay was performed in triplicate, with three independent experiments.

### 2.5 Cross-reactivity analysis

Monoclonal antibody cross-reactivity was assessed by immunodot blotting. Polysaccharide fractions were extracted from VREF E155, *E. faecium* ATCC 19434, *E. faecalis* ATCC 29212, and *Staphylococcus aureus* ATCC 25923 using hot phenol-water extraction. Polysaccharides (1 μg) were spotted onto nitrocellulose membranes, blocked with 5% non-fat milk in PBS-Tween, and incubated with monoclonal antibodies (1 μg/ml) overnight at 4 °C. After washing, the membranes were incubated with HRP-conjugated anti-mouse IgG and developed using a chemiluminescent substrate.

### 2.6 A rabbit model of VREF bacteremia: detailed protocol

#### 2.6.1 Animals and housing

Female New Zealand White (NZW) rabbits (2.5–3.5 kg, *n* = 15) were obtained from a certified supplier (Bioterio, Facultad de Medicina, UNAM) and housed individually in stainless steel cages (60 cm × 60 cm × 45 cm) in a climate-controlled facility (temperature: 20 °C–22 °C, humidity: 50%–60%, 12h light/dark cycle). The rabbits were acclimatized for 14 days prior to experimentation. The rabbits had *ad libitum* access to standard rabbit chow (Harlan Teklad) and sterile water. All experimental procedures were approved by the Institutional Animal Care and Use Committee (Protocol 053/2013, Comités de Investigación y Ética, Facultad de Medicina) and conducted in accordance with Mexican Official Norm NOM-062-ZOO-1999 (https://www.fmvz.unam.mx/fmvz/principal/archivos/062ZOO.PDF).

#### 2.6.2 Experimental groups

Rabbits were randomly assigned to three groups (*n* = 6 per group): Experimental group (VREF + mAb 8AP), infected with VREF + treated with mAb 8AP; Infected/untreated group (VREF + Saline); Control group (Saline Control), inoculated with sterile saline only (no infection).

#### 2.6.3 Infection protocol (Day 0)

Rabbits were anesthetized with ketamine (35 mg/kg IM) and xylazine (5 mg/kg IM) administered into the hind limb musculature. The marginal ear vein was catheterized with a 24-gauge IV catheter (BD Angiocath) after aseptic preparation of the ear with 70% ethanol followed by povidone-iodine solution. At time 0h, the rabbits in experimental and infected/untreated groups received 1 ml of bacterial suspension (1 × 10^9^ CFU) via slow IV injection over 2 min to avoid bolus-related complications. Control group rabbits received 1 ml of sterile saline via the same route. Immediately following infection (within 5 min), the experimental group rabbits received 500 μl of concentrated mAb 8AP (75% v/v, approximately 15 mg total antibody) via IV injection through the same catheter; infected/untreated group rabbits received 500 μl of sterile saline. The catheters were flushed with 0.5 ml of sterile saline, removed, and pressure was applied to the injection site for 2 min to ensure hemostasis. The rabbits were monitored during recovery from anesthesia (30–60 min) in heated recovery cages and returned to individual housing cages once they were fully ambulatory.

#### 2.6.4 Clinical monitoring schedule

The rabbits in each group were monitored by trained personnel blinded to treatment group at the following time points: 0h (baseline, pre-infection), 12h, 24h, 39h, 48h, 72h, and 96h post-infection. At each timepoint, the following parameters were assessed: *body weight:* this was measured using a calibrated digital scale (Ohaus Scout Pro, ± 1 g accuracy). Rabbits were gently restrained in a cloth bag for weighing. *Body temperature:* measured rectally using a digital veterinary thermometer (GLA M750, ± 0.1 °C accuracy) inserted 4–5 cm into the rectum and held for 60 s until the reading stabilized. *Heart rate*: This was measured by cardiac auscultation using a stethoscope placed on the left thorax for 60 s. *Respiratory rate*: This was measured by direct observation of thoracic movements for 60 s while the rabbit was at rest. The counts of two independent observers were averaged. *Clinical signs*: These were assessed using a standardized scoring system (0–3 scale): Lethargy: 0 = normal activity, 1 = mildly reduced activity, 2 = markedly reduced activity; anorexia: 0 = normal food consumption, 1 = reduced consumption, 2 = minimal consumption, 3 = no consumption, piloerection: 0 = absent, 1 = mild, 2 = moderate, 3 = severe and Abnormal posture: 0 = normal, 1 = hunched, 2 = recumbent but responsive, 3 = recumbent and unresponsive. *Blood collection*: 0.5 ml of blood was collected via the marginal ear vein (contralateral to the infection site) into EDTA tubes (BD Vacutainer) for bacterial culture and complete blood counts. Blood cultures were performed by plating 100 μl onto TS and CHROMO agar VRE and incubating at 37 °C for 24–48h. Leukocyte counts were performed using a hematology analyzer.

#### 2.6.5 Humane endpoints

The rabbits were monitored continuously (every 4–6h) for signs of severe distress. Humane endpoints were established *a priori* and included (1) the inability to obtain food or water for >12h, (2) >20% body weight loss from the baseline, (3) severe respiratory distress (respiratory rate >80 breaths/min or labored breathing), (4) hypothermia (<37 °C), (5) seizures or neurological signs, or (6) being unresponsive to external stimuli. Animals meeting any of these criteria were immediately euthanized as described below. Two rabbits (20% of the infected animals, both from infected/untreated) reached humane endpoints at 72h and 84h post-infection and were euthanized prior to the 96h endpoint.

#### 2.6.6 Sacrifice and organ collection (96h)

At 96h post-infection, the surviving rabbits were deeply anesthetized with ketamine (50 mg/kg IM) and xylazine (10 mg/kg IM), followed by euthanasia via IV injection of sodium pentobarbital (150 mg/kg, Euthanex) into the marginal ear vein. Death was confirmed by the absence of a heartbeat and corneal reflexes.

Blood (5–10 ml) was immediately collected into EDTA tubes via cardiac puncture and retained for cytokine analysis. Plasma was separated by centrifugation (2,000 × g, 10 min, 4 °C) and stored at −80 °C until analysis.

A midline ventral incision was performed under aseptic conditions, and the organs (liver, spleen, kidneys, lungs, and heart) were aseptically removed, rinsed in sterile PBS, blotted dry, and weighed on a calibrated analytical balance. Each organ was processed as follows:

*For Bacterial Load Quantification:* one portion of each organ (approximately 0.5–1 g) was placed in a sterile stomacher bag with 9 ml sterile PBS and homogenized using a stomacher (Seward Stomacher 400) at high speed for two minutes. Serial 10-fold dilutions were prepared in sterile PBS, and 100 μl of each dilution was plated in duplicate on TS and CHROMO agar VRE. The plates were incubated at 37 °C for 24–48h, and bacterial colonies were counted. CFU counts were normalized to organ weight and expressed as CFU/g tissue. *For Histopathology*: one portion of each organ was fixed in 10% neutral buffered formalin using a tissue/formalin ratio of 1:10 for 24h–48h at room temperature. The fixed tissues were processed using a Leica TP1020, embedded in paraffin blocks, sectioned at 5 μm thickness using a microtome (Leica RM2125), and mounted on glass slides. The sections were stained with hematoxylin and eosin (H&E) using standard protocols. The slides were examined by a board-certified veterinary pathologist blinded to treatment groups using a light microscope (Olympus BX43). Histopathological lesions were scored on a semi-quantitative scale: 0 = no lesions, 1 = mild, 2 = moderate, and 3 = severe. The parameters assessed included inflammation (neutrophilic and lymphocytic), necrosis, hemorrhage, and architectural disruption.

### 2.7 Cytokine quantification

Plasma cytokine levels were measured using a multiplex bead-based immunoassay.

Magnetic Pearl Panel Kit HCYTOMAG-60K (Millipore, Germany). The panel included IL-1α, IL-1β, IL-4, IL-6, IL-8, IL-10, IL-12p70, IL-13, IL-15, IL-17, TNF-α, IFN-γ, GM-CSF, GRO (CXCL1), IP-10 (CXCL10), MIP-1α (CCL3), Eotaxin (CCL11), and MDC (CCL22).

Briefly, 50 μl of plasma (diluted 1:4 in sample diluent) was incubated with antibody-conjugated magnetic beads for one hour at room temperature with shaking. After washing, detection antibodies were added, and the culture was incubated for 30 min, followed by the addition of streptavidin-phycoerythrin and incubation for 10 min. The beads were washed and resuspended in assay buffer, and the fluorescence was measured using a Luminex 200 instrument (Millipore) with Xponent software. Each sample was analyzed in duplicate.

### 2.8 Statistical analysis

Data are presented as mean ± standard deviation (SD) unless otherwise stated. Data normality was assessed using the Shapiro–Wilk test. Comparisons between two groups were performed using an unpaired Student’s t-test (for normally distributed data) or a Mann–Whitney U test (for non-normally distributed data). Comparisons among three or more groups were performed using one-way ANOVA with Tukey’s *post-hoc* test for multiple comparisons (for normally distributed data) or Kruskal–Wallis test with Dunn’s *post-hoc* test (for non-normally distributed data).

Temporal changes within groups were analyzed using repeated-measures ANOVA with a Bonferroni correction for multiple comparisons. Survival was analyzed using Kaplan–Meier curves with the log-rank (Mantel-Cox) test. Categorical data were analyzed using Fisher’s exact test or the chi-square test as appropriate.

Statistical significance was set at *p* < 0.05 (two-tailed tests). All analyses were performed using GraphPad Prism version 9.0 (GraphPad Software, San Diego, CA) and SPSS version 26.0 (IBM, Armonk, NY).

## 3 Results

### 3.1 Bacterial growth characteristics

**Figure 1:**
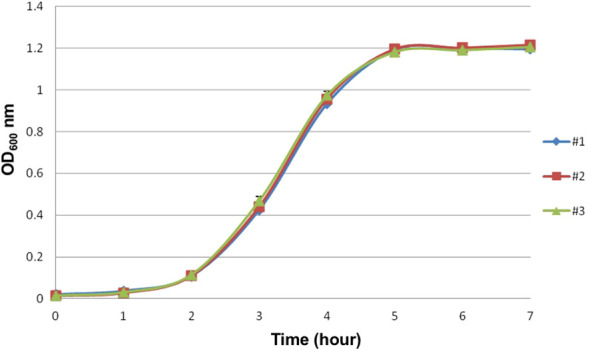
Growth curve of the VREF E155 strain. The assay was performed in triplicate.

*E. faecium* strain E155 exhibited typical enterococcal growth kinetics in BHI broth (**Figure 1**). The 5 × 10^8^ CFU/ml was selected for bacterial harvesting in the infection studies to ensure consistency and replicability.

### 3.2 Bacterial protein profile and characterization

**Figure 2:**
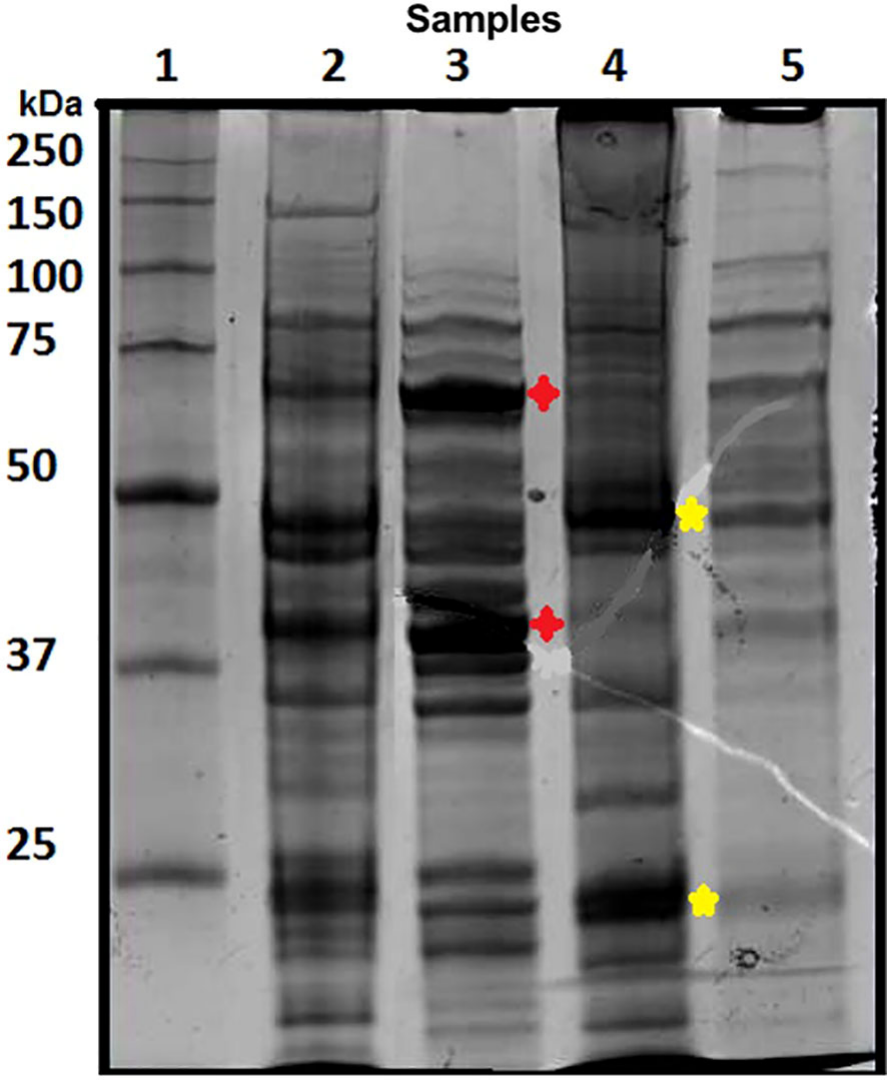
Electrophoretic profile of proteins from the *Enterococcus faecium* VREF E155 strain. Lane 1: Molecular weight marker (MWM); Lane 2: Whole VREF E155 bacteria; Lane 3: Cell wall fraction; Lane 4: Cell membrane fraction; Lane 5: Cytoplasmic fraction. Proteins were separated on a 12% SDS-PAGE gel and stained with colloidal Coomassie blue.

SDS-PAGE analysis of VREF E155 whole-cell lysates revealed a complex protein profile with multiple bands ranging from 10 to 200 kDa (**Figure 2**). Prominent bands were observed at approximately 70, 55, 40, and 30 kDa, consistent with previously reported enterococcal protein profiles. These included putative surface proteins and virulence factors that served as targets to produce monoclonal antibodies.

### 3.3 Monoclonal antibody characterization and opsonophagocytic activity

Nine hybridoma clones producing monoclonal antibodies against VREF E155 were generated: five clones (2AP, 8AP, 9AP, 3BP, and 9BP) produced antibodies against cell wall fractions, and four clones (6 AM, 11AM, 4BM, and 10BM) produced antibodies against membrane fractions (**Table 1**).

**Table 1:** Characterization of monoclonal antibody-producing clones.

Clone	Target fraction	Isotype	ELISA reactivity (OD_450_)	OPA % Killing	Selected for *in-vivo* study
2AP	Cell wall	IgG1	2.45 ± 0.18	68.5 ± 8.2	No
8AP	Cell wall	IgG1	2.78 ± 0.22	75.3 ± 6.5	**Yes**
9AP	Cell wall	IgG3	1.89 ± 0.15	52.4 ± 9.1	No
3BP	Cell wall	IgG1	2.12 ± 0.19	61.2 ± 7.8	No
9BP	Cell wall	IgG3	3.01 ± 0.25	15.8 ± 5.4	No
6AM	Membrane	IgG1	1.95 ± 0.16	72.1 ± 7.3	No
11AM	Membrane	IgG2b	2.34 ± 0.20	70.8 ± 8.6	No
4BM	Membrane	IgG1	1.67 ± 0.14	58.9 ± 9.2	No
10BM	Membrane	IgG3	1.43 ± 0.12	45.3 ± 10.1	No

A rabbit model of acute bacteremia and sepsis caused by vancomycin-resistant *Enterococcus faecium*.

**Figure 3:**
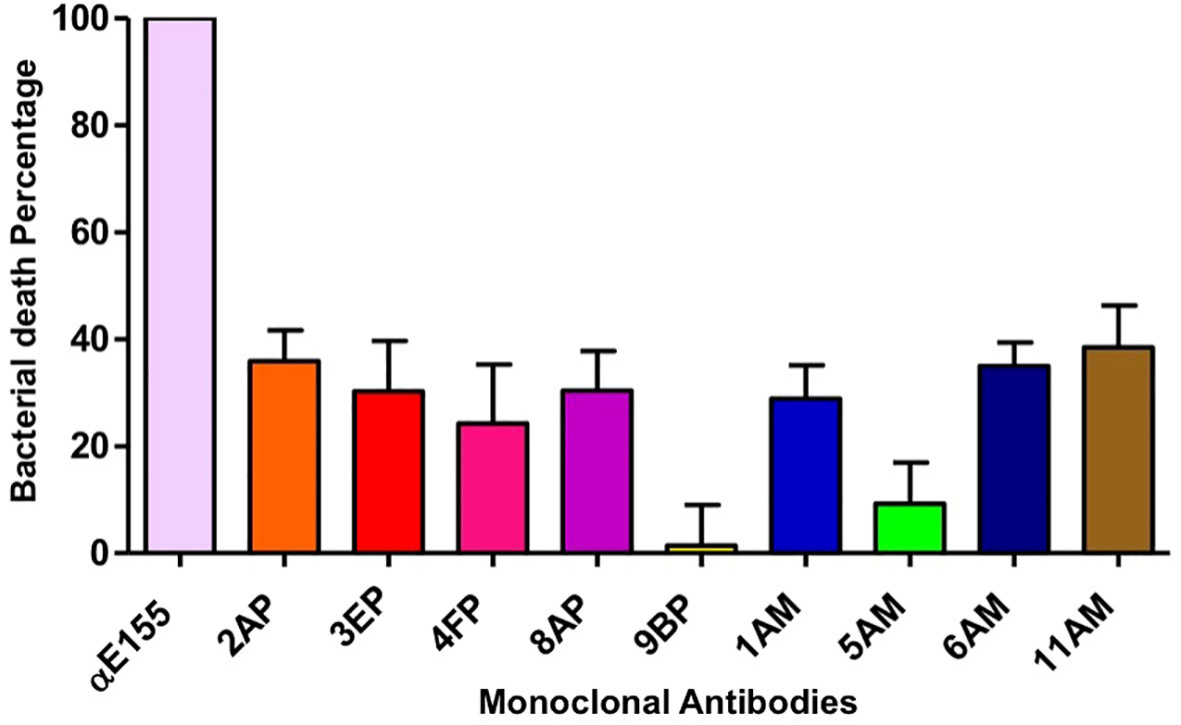
Percent bacterial killing of the VREF E155 strain in the opsonophagocytic assay. The first column represents the positive control using rabbit polyclonal antibodies against the VREF E155 strain. The subsequent nine columns show the results for nine monoclonal antibodies; each tested at a 1:100 dilution. The bar represent the mean ± standard deviation.

Opsonophagocytic assays demonstrated that seven of nine monoclonal antibodies significantly enhanced bacterial killing by human PMNs compared to the no-antibody controls (*p* < 0.01; **Figure 3**). Clone 8AP exhibited the highest opsonophagocytic activity (75.3 ± 6.5% killing), significantly higher than clone 9BP (15.8 ± 5.4%; *p* < 0.001), which showed high ELISA reactivity but poor functional activity. Most of the antibodies were IgG1 or IgG3 isotypes that bind to human Fcγ receptors on phagocytes with high affinity, thereby facilitating opsonization.

Clone 8AP was selected for *in-vivo* studies based on: (1) the highest opsonophagocytic killing activity, (2) the IgG1 isotype (optimal for Fcγ receptor engagement), (3) strong reactivity to cell wall polysaccharides, and (4) cross-reactivity with other enterococcal species.

### 3.4 Cross-reactivity of monoclonal antibodies

**Figure 4:**
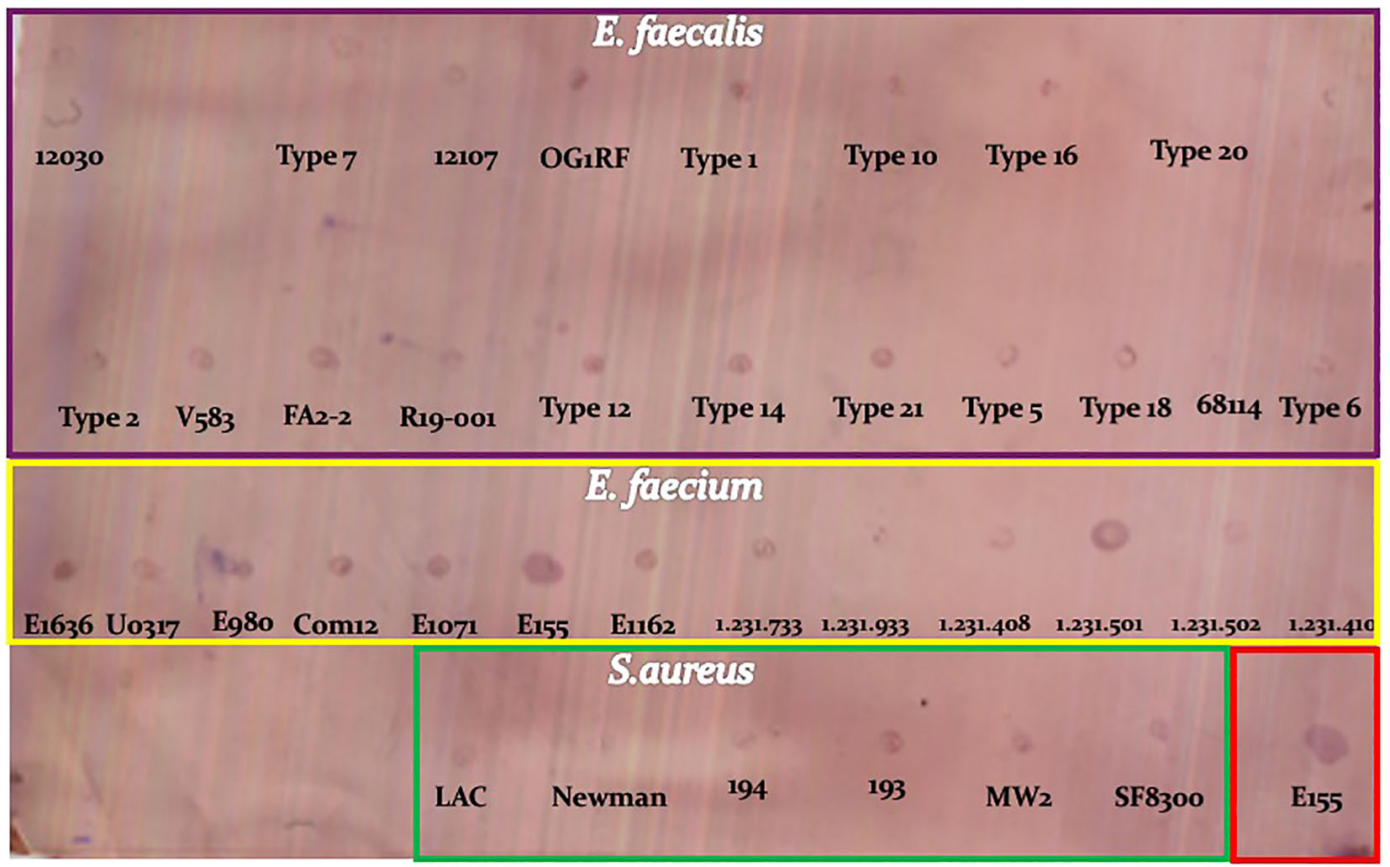
Immune dot blot showing recognition by monoclonal antibody 8AP of the cell wall-associated polysaccharide fraction from *Enterococcus* and *Staphylococcus* species. The purple box highlights the *E. faecalis* strains (*n* = 19), the yellow box indicates the *E. faecium* strains (*n* = 13), the green box shows the *S. aureus* strains (*n* = 6), and the red box marks the control strain VREF E155.

Immunodot blot analysis demonstrated that mAb 8AP recognized polysaccharide antigens from VREF E155, *E. faecalis* ATCC 29212, and *S. aureus* ATCC 25923, indicating cross-reactivity with shared carbohydrate epitopes (**Figure 4**). mAb 6AM and 11AM also showed cross-reactivity, while other clones were strain specific.

### 3.5 Clinical manifestations of VREF bacteremia in rabbits

**Figure 5:**
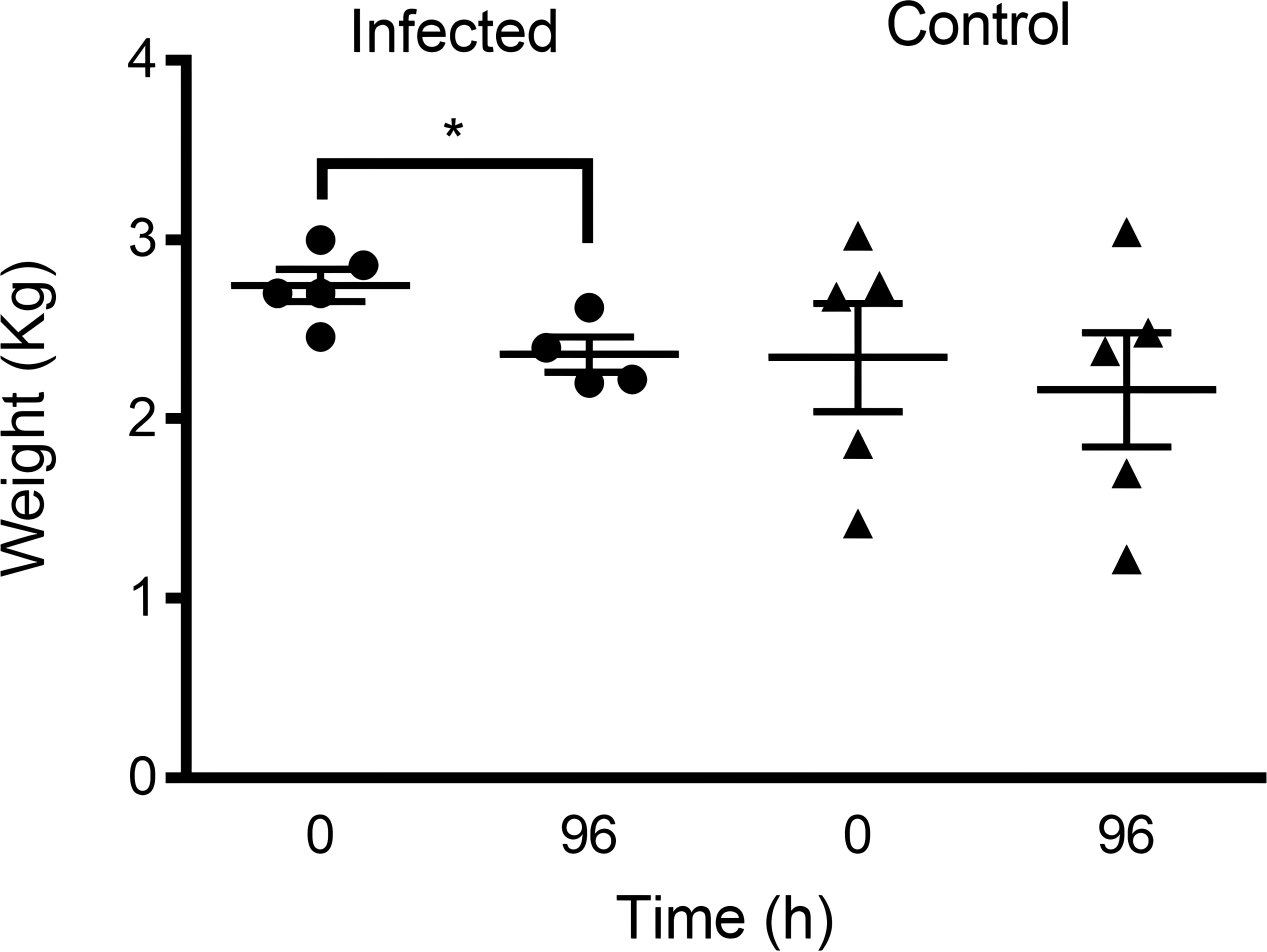
Determination of rabbit body weight in VREF infection model. The center lines represent the mean and error bars represent standard deviation. *p<0.05 is considered significantly different using Mann-Whitney test.

**Figure 6:**
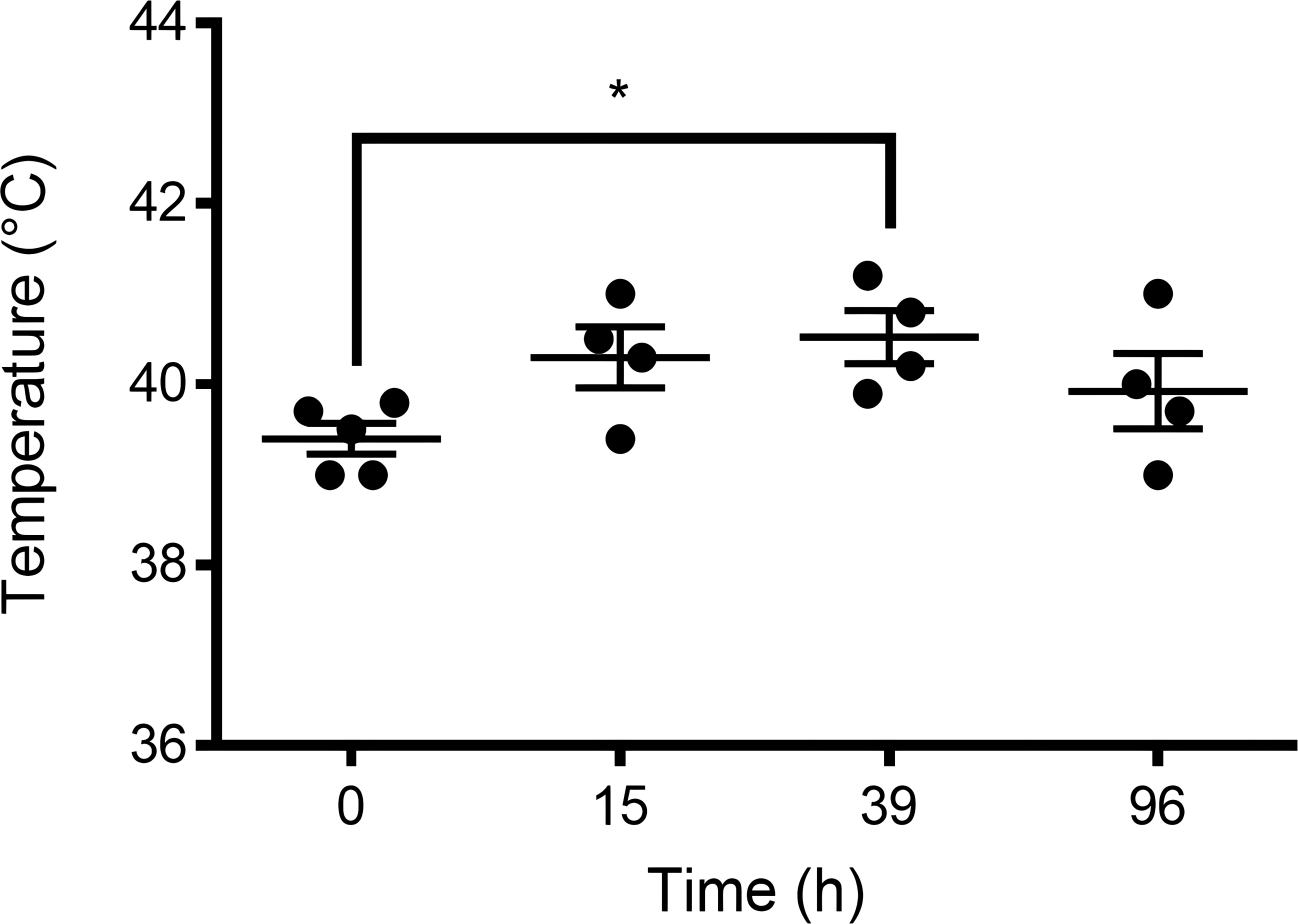
Chronology of body temperature in the rabbit infection model with VREF. Chronology of body temperature in rabbits infected with VREF (1 × 10^9^ CFU/g), reported in °C, with an initial *n* = 5 rabbits. T = 0h marks the beginning of infection, and T = 96h marks the end of monitoring and sacrifice. At 12h there was 20% mortality in the group of infected rabbits. At 39h the feverish peak occurred, which shows a significant difference when compared to *t* = 0. *p* < 0.05 is considered significantly different using Mann-Whitney test. The center lines represent the mean and error bars represent standard deviation. *p<0.05 is considered significantly different using Mann-Whitney test.

All rabbits in the infected and experimental group (*n* = 10) developed clinical signs of bacteremia within 12h–24h post-infection, whereas the control group (*n* = 5) remained healthy throughout the study period. *Body weight*: infected rabbits exhibited progressive weight loss beginning at 24h post-infection (**Figure 5**; **Table 2**). By 96h, untreated infected rabbits (infected group: VREF + Saline) had lost 19.2 ± 5.1% of their initial body weight compared to only 1.6 ± 2.1% in the control group (*p* < 0.001). The mAb 8AP-treated rabbits (experimental/treated group) showed significantly attenuated weight loss (10.7 ± 4.2%; *p* < 0.05 *vs*. the infected group), indicating preserved metabolic function and reduced disease severity. *Body temperature*: all infected rabbits developed sustained fever (>40 °C) beginning at 12h–24h post-infection and persisting through 96h, meeting the SIRS temperature criteria (**Figure 6**, **Table 3**). Baseline temperatures were normal in all groups (38.6–38.8 °C). Peak temperatures were observed at 39h post-infection (40.5 ± 0.6 °C in the experimental group; 40.7 ± 0.5 °C in the infected/untreated group). There were no significant differences in temperature between mAb-treated and untreated infected groups at any time point (*p* > 0.05), suggesting that fever response was independent of antibody treatment in this model. *Clinical Signs*: Infected rabbits developed lethargy (70.0 ± 14.5% of infected rabbits scored ≥ 2), anorexia, piloerection, and a hunched posture by 24–48h post-infection. Control rabbits maintained normal activity, appetite, and grooming behavior throughout the study (**Supplementary Table 1**). *Mortality*: Two rabbits from infected/untreated (VREF + Saline, 20% mortality) reached humane endpoints and were euthanized at 72h and 84h post-infection due to severe clinical deterioration (>20% weight loss, severe lethargy, hypothermia). No mortality occurred in the experimental group (VREF + mAb 8AP) or in the control group during the entire duration of the experiment (96h). The difference in mortality between experimental individuals did not reach statistical significance at the 0.05 level (*p* = 0.14, Fisher’s exact test), likely due to the small sample size. *Bacteremia*: Blood cultures were positive for Gram-positive cocci in all infected rabbits (10/10, 100%) at all time points from 12h through 96h, confirming sustained bacteremia. Bacterial loads in the blood ranged from 10³ to 10^5^ CFU/mL. Control rabbits had sterile blood cultures at all timepoints.

**Table 2:** Changes in body weight over time (kg).

Timepoint	Group 1: VREF + mAb 8AP (*n* = 5)	Group 2: VREF + Saline (*n* = 5)	Group 3: Saline control (*n* = 5)	*P*-value
0h (baseline)	3.18 ± 0.25	3.12 ± 0.31	3.22 ± 0.31	0.812
24h	3.08 ± 0.27	2.98 ± 0.35	3.20 ± 0.29	0.342
48h	2.95 ± 0.28	2.79 ± 0.42	3.19 ± 0.30	0.078
72h	2.88 ± 0.29	2.63 ± 0.45*	3.18 ± 0.28	0.024
96h	2.84 ± 0.30	2.52 ± 0.48 *	3.17 ± 0.27	0.012
% Change (0–96h)	−10.7 ± 4.2%	−19.2 ± 5.1%*	−1.6 ± 2.1%	< 0.001

Data are presented as Mean ± SD. Statistical comparisons were performed using repeated measures ANOVA with Tukey’s post-hoc test. *p < 0.05 vs. control group. Two rabbits from the infected but untreated group were euthanized at 72h and 84h due to reaching humane endpoints; their last recorded weights were carried forward for the 96h analysis.

**Table 3:** Body temperature at baseline and 96h.

Group	0h temperature (°C)	96h temperature (°C)	Change (°C)	*P*-value (within group)
Group 1: VREF + mAb 8AP (*n* = 5)	38.8 ± 0.4	40.0 ± 0.6	+1.2 ± 0.7	< 0.001
Group 2: VREF + Saline (*n* = 5)	38.6 ± 0.4	40.2 ± 0.6	+1.6 ± 0.7	< 0.001
Group 3: Saline control (*n* = 5)	38.6 ± 0.3	38.8 ± 0.4	+0.2 ± 0.3	0.156

Data are presented as Mean ± SD. Within-group comparisons were performed using a paired t-test; between-group comparisons were performed using an unpaired t-test.

### 3.6 Systemic bacterial dissemination and organ pathology

#### 3.6.1 Macroscopic findings

**Figure 7:**
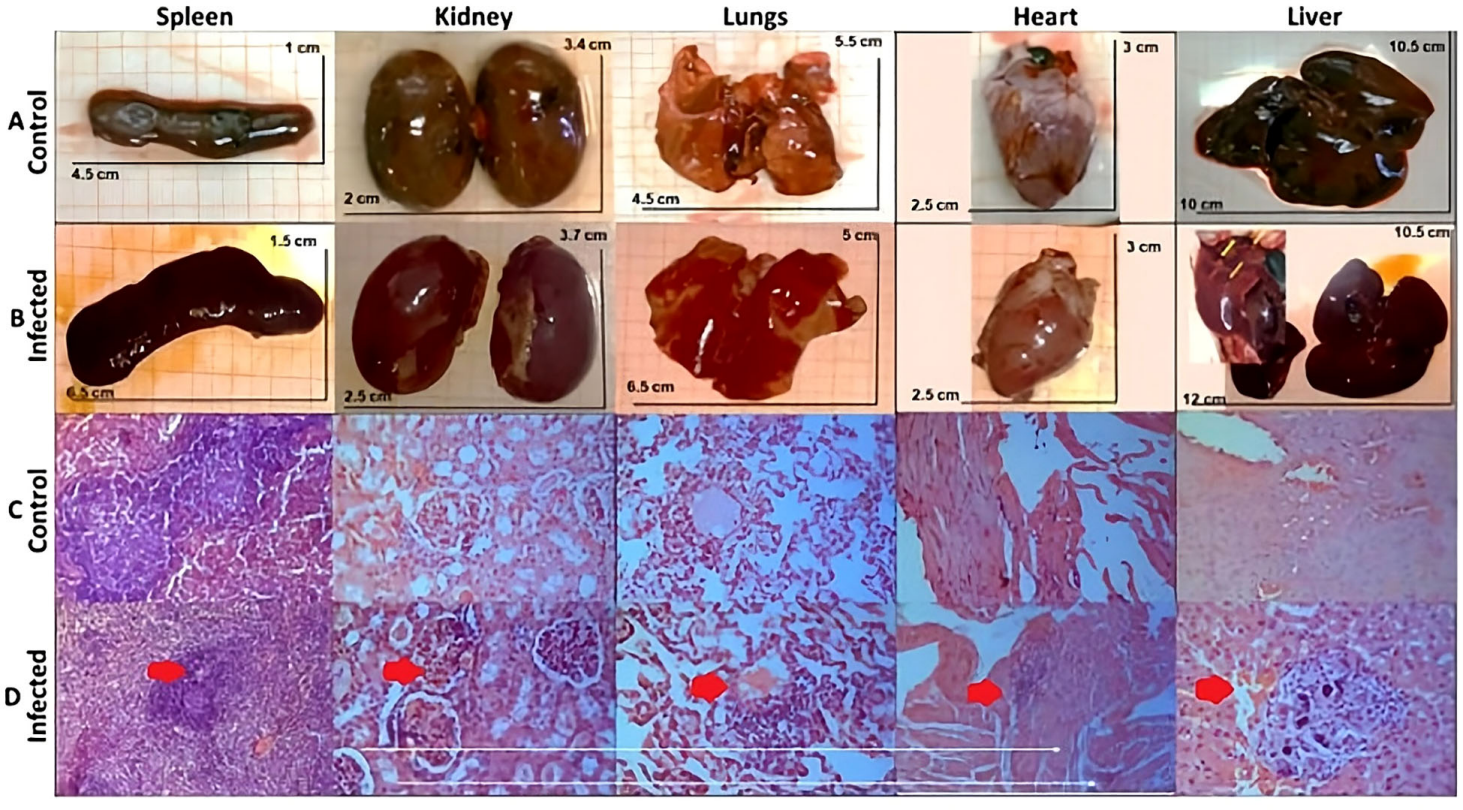
Morphology and histology of organs from the rabbit infection model with VREF. NZW rabbits infected with 1 × 10^9^ CFU/g intravenous route. They were sacrificed at 96h, and their organs were evaluated. Organs from control group rabbits **(A)** and infected with VREF **(B)**. Histological section of the organ from the control group **(C)** and from the VREF-infected group **(D)** stained with hematoxylin-eosin. 40× magnification.

**Figure 8:**
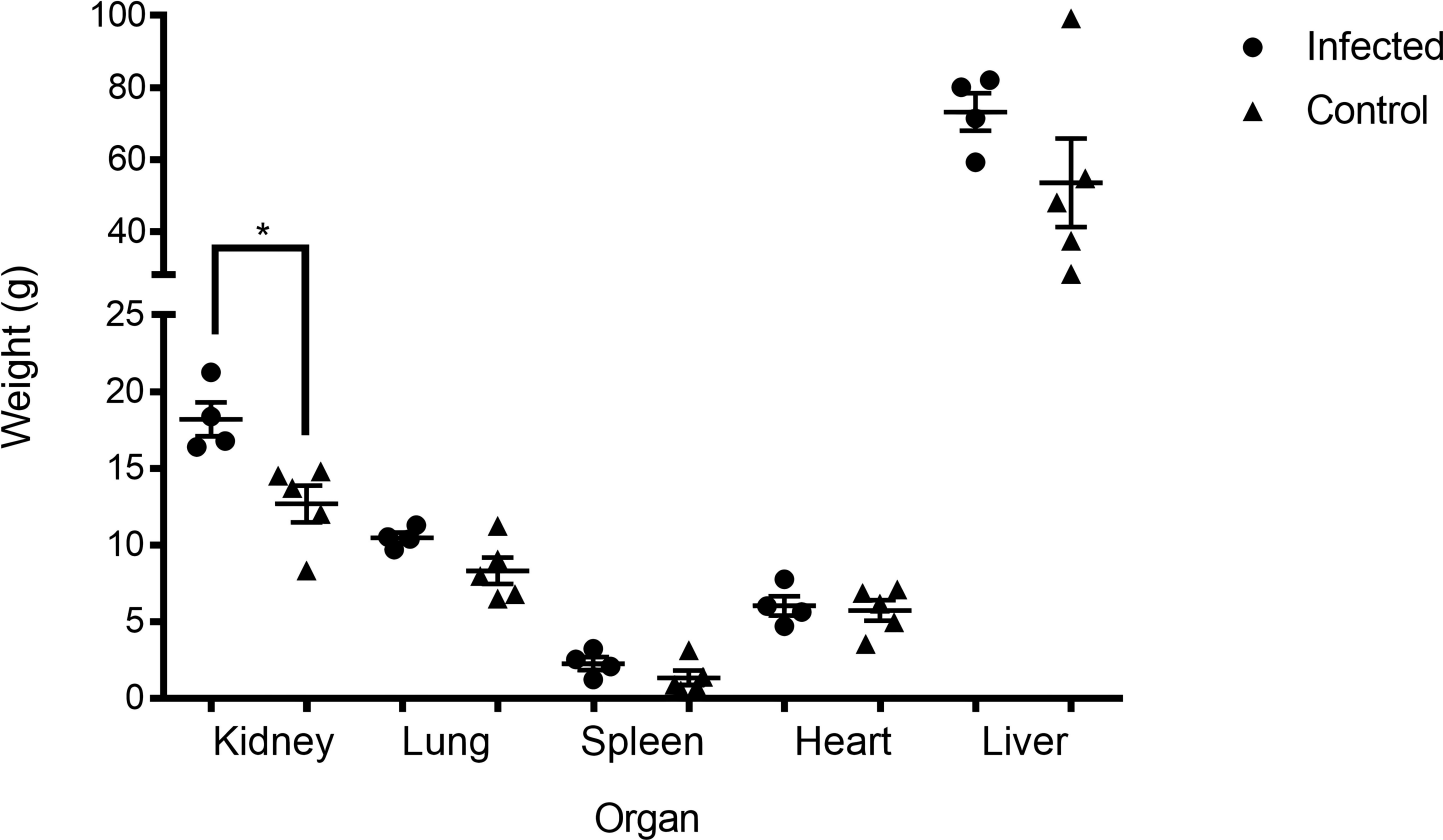
Weight differences in organs infected with EFRV versus control group. Weight in grams of kidneys, lungs, spleen, heart, and liver of the infected group (IV route, 1 × 10^9^ CFU/g, 96h) and the control group (IV route, SSI 1 ml, 96h), significant difference observed between groups in the kidney. *p* < 0.05 is considered significantly different using Mann–Whitney test. The center lines represent the mean and error bars represent standard deviation. *p<0.05 is considered significantly different using Mann-Whitney test.

Gross examination at necropsy revealed marked organomegaly and pathological changes in the infected rabbits (**Figures 7A, B**). The liver exhibited the most pronounced changes, with visible necrotic foci (yellow–white lesions, 2–5 mm in diameter) scattered throughout the parenchyma and hepatomegaly. The spleen was enlarged and dark red with prominent white nodules. The kidneys showed cortical petechiae and mild enlargement. The lungs had multifocal areas of consolidation and hemorrhage. The heart appeared normal in most cases, although mild pericardial effusion was noted in 2 of 10 infected rabbits. Organ weights were significantly higher in infected rabbits than in controls (**Figure 8**). Kidneys (infected: 17.7 ± 0.9 g *vs*. control: 12.4 ± 1.2 g, *p* < 0.001), lungs (infected: 10.0 ± 0.3 g *vs*. control: 8.1 ± 0.7 g, *p* = 0.052), and livers (infected: 73.4 ± 4.0 g *vs*. control: 56.0 ± 12.0 g, *p* = 0.078). The liver and lungs were not significantly different.

#### 3.6.2 Microscopic findings

Histopathological examination revealed severe pathological changes in multiple organs of infected rabbits (**Figures 7C, D**; **Table 4**).

**Table 4:** Histopathological severity scores in infected rabbits.

Organ	No lesions (0)	Mild (1)	Moderate (2)	Severe (3)	Mean Score ± *SD*
Liver	0/10 (0%)	0/10 (0%)	3/10 (30%)	7/10 (70%)	2.70 ± 0.46
Spleen	0/10 (0%)	2/10 (20%)	4/10 (40%)	4/10 (40%)	2.20 ± 0.79
Kidney	0/10 (0%)	4/10 (40%)	6/10 (60%)	0/10 (0%)	1.60 ± 0.52
Lung	0/10 (0%)	5/10 (50%)	5/10 (50%)	0/10 (0%)	1.50 ± 0.53
Heart	4/10 (40%)	6/10 (60%)	0/10 (0%)	0/10 (0%)	0.60 ± 0.52

Histopathological severity was scored on a scale of 0–3 by a blinded veterinary pathologist. Data are presented as frequency distributions and means ± SD. All control rabbits (n = 5) scored 0 (no lesions) in all organs.

*Liver:* The liver exhibited the most severe pathology. All infected rabbits (10/10) showed multifocal to coalescing areas of hepatocellular necrosis with extensive neutrophilic infiltration (suppurative inflammation). The necrotic foci ranged from 0.5 to 3 mm in diameter and were characterized by loss of hepatocyte architecture, karyolysis, and infiltration by degenerate neutrophils. Gram staining confirmed the presence of Gram-positive cocci within necrotic areas and phagocytosed by neutrophils. Periportal inflammation and sinusoidal congestion were also prominent, as shown in **Table 4**. *Spleen:* The splenic architecture was disrupted, with depletion of lymphoid follicles and expansion of red pulp. Multifocal microabscesses (100–500 μm in diameter) composed of neutrophils and necrotic debris were present in 8/10 infected rabbits. Hemosiderin deposition was increased, indicating hemolysis (**Table 4**). *Kidneys*: Acute tubular necrosis, glomerulonephritis, and interstitial nephritis were present. The tubular epithelial cells exhibited vacuolar degeneration, loss of brush border, and sloughing into the tubular lumens. Glomeruli showed hypercellularity and capillary congestion. Interstitial inflammation was predominantly neutrophilic, with scattered lymphocytes (**Table 4**). *Lungs*: Exhibited bronchopneumonia with neutrophilic infiltration of alveolar spaces, bronchiolar lumens, and interstitium. Alveolar septa were thickened with edema and inflammatory cells. Multifocal hemorrhage and fibrin deposition were present (**Table 4**). *Heart*: Cardiac pathology was less severe and less consistent than in other organs. Myocarditis, characterized by focal inflammatory infiltrates (neutrophils and lymphocytes), was present in 6 of 10 infected rabbits. Myocyte degeneration and necrosis were rare (**Table 4**). The control rabbits group showed normal histological architecture in all organs, with no inflammation, necrosis, or bacterial presence.

#### 3.6.3 Bacterial load in organs

**Figure 9:**
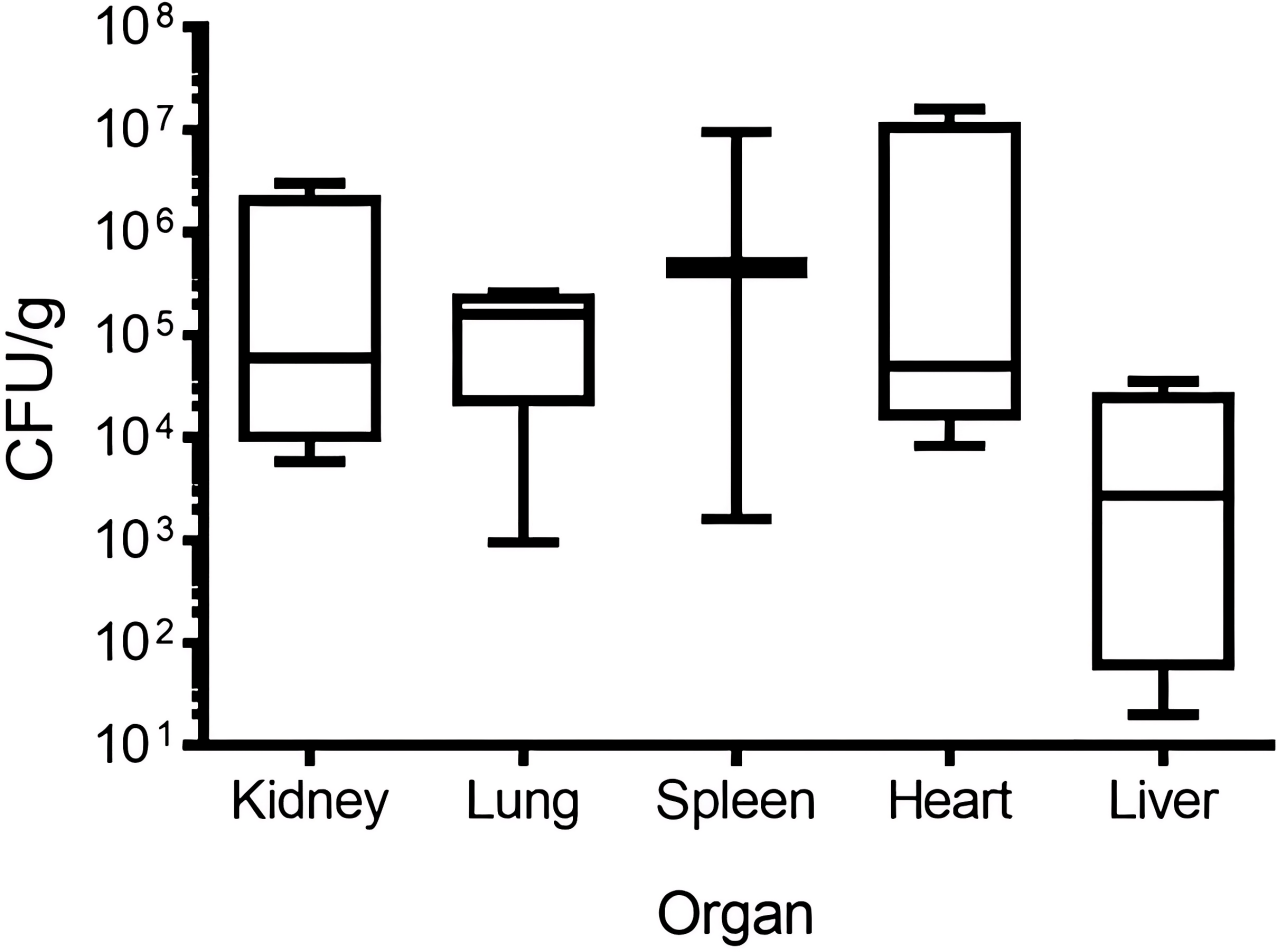
CFU/g of organ from rabbit infected model with VREF From the organs from NZW rabbits (*n* = 4) infected with 1 × 10^9^ CFU/g intravenous route. They were disaggregated, and serial dilutions were made for the quantification of the CFU. Each CFU value was divided by the weight in g of each organ. The center lines represent the mean and error bars represent standard deviation.

Quantitative bacterial cultures confirmed systemic dissemination of VREF to all examined organs (**Figure 9**). Bacterial loads varied significantly among organs: spleen: 10^5^·^6^ ± 10¹·² CFU/g (the highest burden when normalized for organ weight); heart: 10^4^·^5^ ± 10²·^7^ CFU/g; kidney: 10^4^·^5^ ± 10²·^0^ CFU/g; lung: 10^5^·^0^ ± 10^0^·^8^ CFU/g; and liver: 10³·^5^ ± 10²·^7^ CFU/g (the lowest burden despite the most severe pathology).

The spleen had the highest bacterial load after normalization for organ size and weight, consistent with its role as a major reticuloendothelial organ and the site of bacterial sequestration. Interestingly, the liver had the lowest bacterial burden despite exhibiting the most severe histopathological lesions, suggesting that hepatic damage may result from intense inflammatory responses and immune-mediated injury rather than solely from bacterial load. No bacteria were recovered from the organs of control rabbits.

### 3.7 Impact of mAb 8AP treatment on clinical parameters

We systematically monitored clinical parameters throughout the 96h experimental period to assess the effect of mAb 8AP on the course of the disease. These parameters were selected to assess systemic inflammatory response syndrome (SIRS) criteria and overall disease severity, providing translational relevance to human sepsis. *Heart rate*: VREF-infected rabbits showed significantly higher heart rates than the controls beginning at 24h post-infection (**Table 5**). By 48h, the untreated, infected rabbits (VREF/untreated) exhibited marked tachycardia (245 ± 22 bpm) compared to the controls (183 ± 12 bpm, *p* < 0.001), reflecting cardiovascular stress associated with systemic infection and the release of inflammatory mediators. Notably, rabbits treated with mAb 8AP (the experimental group) demonstrated significantly lower heart rates than the infected group (215 ± 18 bpm at 48h, *p* < 0.05), and this protective effect became more pronounced by 96h (198 ± 15 bpm in the mAb-treated group *vs*. 238 ± 20 bpm in the untreated group; *p* < 0.01). *Respiratory rate*: similar patterns were observed for respiratory rate. At 48h post-infection, untreated, infected rabbits had respiratory rates of 72 ± 10 breaths/min, compared with 43 ± 5 breaths/min in controls (*p* < 0.001). The mAb 8AP-treated rabbits exhibited significantly lower respiratory rates than the untreated rabbits (58 ± 8 breaths/min at 48h, *p* < 0.05), with further improvement by 96h (51 ± 7 *vs*. 68 ± 9 breaths/min, *p* < 0.01). *Body Weight*: The final body weights at 96h were significantly different between the treated (2.84 ± 0.30 kg) and untreated (2.52 ± 0.48 kg) infected groups (*p* < 0.05). *Temperature:* All infected rabbits developed sustained fever (>40 °C) beginning at 12–24h post-infection and persisting through 96h, meeting SIRS temperature criteria. There were no significant differences in temperature between mAb-treated and untreated infected groups at any time point (*p* > 0.05). Both infected groups maintained temperatures of 40.0 °C–40.3 °C at 96h compared to 38.8 °C ± 0.4 °C in the controls (*p* < 0.001). *Leukocyte count*: total white blood cell counts were highly variable, and there were no statistically significant differences between groups or time points. Baseline leukocyte counts were 6.5–8.2 × 10³/μl across all groups. At 96h, the counts ranged from 5.8–12.5 × 10³/μl in infected rabbits to 6.8–8.5 × 10³/μl in controls (*p* = 0.28). This may reflect the complex dynamics of leukocyte mobilization, margination, and tissue infiltration during systemic infection.

**Table 5:** Clinical parameters across groups and timepoints.

Parameter	Time point	Experimental: VREF + mAb 8AP (*n* = 5)	Infected/untreated: VREF + Saline (*n* = 5)	Saline control (*n* = 5)	*P*-value
Heart rate (bpm)	0h	185 ± 12	182 ± 15	180 ± 10	0.782
	48h	215 ± 18*	245 ± 22*†	183 ± 12	< 0.001
	96h	198 ± 15*	238 ± 20*†	185 ± 11	< 0.001
Respiratory rate (breaths/min)	0h	42 ± 5	40 ± 6	41 ± 4	0.823
	48h	58 ± 8*	72 ± 10*†	43 ± 5	< 0.001
	96h	51 ± 7*	68 ± 9*†	42 ± 6	< 0.001
Body weight (kg)	0h	3.18 ± 0.25	3.12 ± 0.31	3.22 ± 0.31	0.812
	48h	2.95 ± 0.28	2.79 ± 0.42	3.19 ± 0.30	0.078
	96h	2.84 ± 0.30*	2.52 ± 0.48*†	3.17 ± 0.27	0.012
Temperature (°C)	0h	38.8 ± 0.4	38.6 ± 0.4	38.6 ± 0.3	0.692
	48h	40.1 ± 0.7*	40.3 ± 0.5*	38.7 ± 0.4	< 0.001
	96h	40.0 ± 0.6*	40.2 ± 0.6*	38.8 ± 0.4	< 0.001

Data are presented as Mean ± SD. Statistical comparisons were performed using repeated measures ANOVA with Tukey’s post-hoc test.*p < 0.05 versus control; †p < 0.05 versus experimental (VREF + mAb 8AP).

### 3.8 Quantification of plasma cytokine levels

We quantified plasma levels of 16 cytokines at 96h post-infection using a multiplex bead-based immunoassay to characterize the systemic inflammatory response to VREF infection and assess the immunomodulatory effects of mAb 8AP treatment (**Table 6**).

**Table 6:** Plasma cytokine levels at 96h post-infection (pg/ml).

Cytokine	Experimental group: VREF + mAb 8AP (*n* = 5)	Infected/untreated: VREF + Saline (*n* = 5)	Control: saline control (*n* = 5)	*P*-value
IL-6	1,920 ± 980	2,850 ± 1,240	85 ± 42	< 0.001*
IL-8	2,680 ± 1,320	3,420 ± 1,580	120 ± 65	< 0.001*
IL-10	1,520 ± 690	1,650 ± 780	95 ± 52	< 0.001*
IL-1β	680 ± 385	845 ± 420	45 ± 28	< 0.01*
IL-1α	185 ± 125	220 ± 145	22 ± 15	0.024*
IL-4	240 ± 165	280 ± 185	35 ± 22	0.045*
IL-12p70	125 ± 82	145 ± 95	18 ± 12	0.038*
IL-17	95 ± 68	115 ± 75	12 ± 8	0.042*
GM-CSF	320 ± 180	385 ± 210	42 ± 28	0.012*
IP-10	1,150 ± 620	1,380 ± 740	125 ± 68	< 0.01*
MIP-1α	580 ± 320	685 ± 380	68 ± 35	0.008*
Eotaxin	420 ± 240	485 ± 280	52 ± 30	0.018*
MDC	195 ± 115	225 ± 135	28 ± 18	0.028*
IL-13	85 ± 58	98 ± 65	15 ± 10	0.052
IL-15	72 ± 48	85 ± 55	12 ± 8	0.046*
IL-10/IL-6 ratio	0.79 ± 0.25	0.58 ± 0.18	1.12 ± 0.35	0.015*

Data presented as Mean ± SD. Statistical comparisons were performed using one-way ANOVA with Tukey’s post-hoc test. *p < 0.05 for overall group effect. TNF-α and IFN-γ levels were below detection limits in all samples.

*Pro-inflammatory cytokines*: VREF-infected rabbits exhibited significant increases in key pro-inflammatory cytokines compared to the controls. IL-6, a central mediator of the acute phase response and an established biomarker of sepsis severity, was markedly elevated in infected animals (infected/untreated: VREF + saline: 2,850 ± 1,240 pg/ml; experimental group: VREF + mAb: 1,920 ± 980 pg/ml) compared to the controls (85 ± 42 pg/ml, *p* < 0.001). The lower IL-6 levels in mAb-treated animals, although not statistically significant (*p* = 0.089), suggest a trend toward reduced inflammatory burden. *IL-8 (CXCL8)*, a potent neutrophil chemoattractant, was also significantly elevated in infected rabbits (infected/untreated: 3,420 ± 1,580 pg/ml; experimental group: 2,680 ± 1,320 pg/ml) compared to the controls (120 ± 65 pg/ml, *p* < 0.001). The high IL-8 levels were correlated with the extensive neutrophilic infiltration observed in the histopathological analysis, particularly in the liver. *IL-1β*, a key pyrogenic cytokine and activator of endothelial cells, was increased in infected animals (iInfected/untreated group: 845 ± 420 pg/ml; experimental group: 680 ± 385 pg/ml) compared to the controls (45 ± 28 pg/ml, p < 0.01), consistent with the sustained fever observed in infected rabbits. *Anti-inflammatory cytokines*: IL-10, the principal anti-inflammatory cytokine, was concurrently elevated in infected rabbits (Infected/untreated: 1,650 ± 780 pg/ml; Experimental group: 1,520 ± 690 pg/ml) compared to the controls (95 ± 52 pg/ml, *p* < 0.001), indicating activation of compensatory anti-inflammatory mechanisms. The IL-10/IL-6 ratio in infected animals (approximately 0.58–0.79) indicated a predominantly pro-inflammatory state, consistent with the acute phase of sepsis. In human sepsis, IL-10/IL-6 ratios < 1.0 are associated with ongoing inflammation, whereas ratios > 1.0 may indicate transition to a compensatory anti-inflammatory phase. *Other cytokines*: IL-4, a Th2-associated cytokine, showed modest elevation, with broad confidence intervals (infected/untreated: 280 ± 185 pg/ml; experimental: 240 ± 165 pg/ml; control group: 35 ± 22 pg/ml), indicating high inter-individual variation. Similarly, IL-1α, IL-12p70, IL-17, GM-CSF, and IP-10 showed detectable but variable levels across infected animals. The chemokines MIP-1α, eotaxin, and MDC were also elevated in the infected rabbits.

## 4 Discussion

This study has successfully established a novel rabbit model of acute VREF bacteremia that accurately replicates key clinical and pathological features of human infection. The model demonstrated several important characteristics: (1) consistent establishment of bacteremia following intravenous inoculation with 1 × 10^9^ CFU, (2) systemic bacterial dissemination to multiple organs, (3) fulfillment of multiple SIRS criteria, (4) significant mortality (20% at 96h), and (5) prominent hepatic pathology with necrosis and suppurative inflammation.

### 4.1 Rationale for monoclonal antibody generation

In this study, nine clones of the VREF E155 strain producing monoclonal antibodies against the cell wall (five clones) and membrane (four clones) were generated. Knowledge of the isotype of mAbs is of interest, since the antibodies are intended to be opsonizing agents that stimulate phagocytosis and induce bacterial death by recognizing their constant region (Fc) via neutrophil receptors (FcγR). The main phagocytic receptor (FcγRI) on neutrophils recognizes the IgG1 and IgG3 antibody isotypes in humans and the IgG2a and IgG2b isotypes in mice (Rakita et al., 2000; Abbas and L. A. H., 2008); therefore, it is more efficient at stimulating bacterial phagocytosis. According to our results, most of the mAbs expressed the IgG1 and IgG3 isotypes, indicating that they are optimal for Fc receptor engagement.

The induction of bacterial death by the mAb was evaluated using the opsonophagocytosis assay, a marker of a protective immune response against bacterial pathogens. Of the five mAbs generated against the cell wall of VREF E155, four induced significant bacterial death. Although clone 9BP produced the mAbs with the highest reactivity to the cell wall fraction in the ELISA assay, it was unable to induce bacterial death, possibly because it recognized an abundant cell wall antigen that did not confer opsonization (Arduino et al., 1994). Of the four mAbs generated from the membrane fraction, three induced high percentages of bacterial death, implying that the mAbs recognized immunogenic structures associated with or anchored to the bacterial cell wall.

Previous research has demonstrated the presence of polysaccharide structures that confer phagocytosis resistance in encapsulated enterococcal strains. Therefore, the generation of antibodies against these structures enables phagocytosis of the bacteria (Arduino et al., 1994; Huebner et al., 2000; Rakita et al., 2000). In this study, we determined that mAbs from clones 8AP, 6AM, and 11AM recognized polysaccharides associated with the bacterial cell wall, with mAb 8AP selected for therapeutic evaluation due to its superior opsonophagocytic activity and cross-reactivity with other enterococcal species and *S. aureus*.

### 4.2 Rabbit model justification and characteristics

NZW rabbits were chosen as an appropriate model due to their intermediate size, phylogenetic proximity to primates (compared to rodents), and genetic diversity, making them suitable candidates for extrapolating the results to humans (Heinz et al., 2003; Bosze and H. L. M., 2006; Vaure and Liu, 2014; Neves et al., 2015; Peng and K. J. H. K., 2015; Pinheiro et al., 2016; Goh et al., 2017; Esteves et al., 2018; Soares et al., 2022). Rabbits have been previously used to study infectious diseases, including VREF-related endocarditis, but this study focused on acute bacteremia without the involvement of medical interventions (Lefort et al., 2003; Rabinowitz et al., 2005; Pontikis et al., 2013; Chambers et al., 2016; Castañeda et al., 2021; Soares et al., 2022; Patricia V. Turner, 2025). We implemented a therapeutic approach using monoclonal antibodies with high titers and cross-reactivity against *E. faecalis* and *S. aureus*.

Intravenous infection with 1 × 10^9^ CFU of VREF successfully induced acute bacteremia in all rabbits (Lefort et al., 2003). Clinical manifestations included weight loss, fever, and 20% mortality. The high bacterial load required to establish infection in immunocompetent hosts highlights the opportunistic nature of VREF, which predominantly affects immunocompromised hosts. Still, VREF can cause severe infections in immunocompetent individuals exposed to high inoculum levels. This observation demonstrates the pathogen’s ability to overwhelm host defenses under certain conditions, resulting in systemic dissemination and tissue damage.

### 4.3 Systemic dissemination and organ-specific pathology

Our findings revealed that VREF was distributed across multiple organs, including the kidneys, lungs, spleen, heart, and liver, indicating systemic dissemination of the pathogen. This widespread bacterial colonization is consistent with the behavior of highly invasive enterococcal strains and reflects their capacity to translocate across epithelial barriers and evade immune clearance mechanisms. The systemic nature of the infection highlights the clinical relevance of VREF as a cause of severe, disseminated disease, particularly in settings of high bacterial burden or compromised host defenses (Arias and Murray, 2012; Gilmore et al., 2014; Fiore et al., 2019).

Among the affected organs, the liver exhibited the most pronounced pathological lesions. This observation is particularly significant given the liver’s central role in both innate immune surveillance and the clearance of circulating pathogens. Hepatic macrophages (Kupffer cells) are among the first line of defense against bloodstream infections, and their activation is critical for initiating systemic immune responses. The presence of extensive hepatic lesions suggests that VREF may impair or overwhelm these local immune mechanisms, contributing to sustained bacteremia and systemic inflammation. These findings align with previous studies demonstrating that organ-specific immune responses, particularly in the liver and spleen, are pivotal for determining the outcome of systemic bacterial infections (Lefort et al., 2003; Goh et al., 2017).

Interestingly, the liver had the lowest bacterial burden despite exhibiting the most severe histopathological lesions, suggesting that hepatic damage may result from intense inflammatory responses and immune-mediated injury rather than solely from the bacterial load. This immunopathology occurs when the host immune response causes collateral tissue damage while attempting to clear the infection. The observed organ tropism and associated pathology support the need for therapeutic strategies that not only target bacterial viability but also enhance host immune function and tissue protection.

### 4.4 Clinical relevance of physiological parameters

The clinical parameter data provide crucial validation that our rabbit model accurately simulates the pathophysiology of human sepsis. According to the established definition of Systemic Inflammatory Response Syndrome (SIRS), patients must meet at least two of four criteria: temperature > 38 °C or < 36 °C, heart rate > 90 bpm (in humans; >150 bpm in rabbits adjusted for body size), respiratory rate > 20 breaths/min (in humans; >60 breaths/min in rabbits), or white blood cell count abnormalities (Kathleen Pritchett-Corning et al., 2011; Patricia V. Turner, 2025). Our VREF-infected rabbits consistently met multiple SIRS criteria (elevated temperature, heart rate, and respiratory rate), validating the clinical relevance of this model.

The significantly elevated heart rate observed in VREF-infected rabbits reflects cardiovascular stress associated with systemic infection and the release of inflammatory mediators. Tachycardia in sepsis results from multiple mechanisms: the direct effects of inflammatory cytokines (particularly IL-1β and IL-6 (Vella et al., 2025)) on cardiac pacemaker cells, compensatory responses to decreased systemic vascular resistance, and increased metabolic demand. The reduction in heart rate following mAb 8AP treatment suggests that the antibody therapy mitigated cardiovascular strain, potentially through reduction of bacterial load and the associated inflammatory signaling. This finding is clinically significant, as persistent tachycardia in septic patients is associated with poor outcomes and increased mortality.

Similarly, the marked elevation in respiratory rate in infected animals indicates compensatory respiratory responses to metabolic acidosis and systemic inflammation, two hallmarks of sepsis (Bosze and H. L. M., 2006; Kathleen Pritchett-Corning et al., 2011; Peng and K. J. H. K., 2015; Esteves et al., 2018; Patricia V. Turner, 2025). Tachypnea in sepsis compensates for metabolic acidosis by increasing CO_2_ elimination and represents a respiratory response to lactic acidosis generated by tissue hypoperfusion. The significant reduction in respiratory rate in the mAb 8AP-treated animals suggests an improved metabolic status and a reduced inflammatory burden, indicating that the antibody therapy may improve tissue perfusion and reduce anaerobic metabolism.

The progressive weight loss in infected rabbits reflects the catabolic state induced by severe infection (Kathleen Pritchett-Corning et al., 2011; Esteves et al., 2018; Patricia V. Turner, 2025), driven by inflammatory cytokine-mediated breakdown of muscle protein, anorexia, and an increased metabolic rate. Weight loss in sepsis is a well-established clinical phenomenon associated with poor prognosis. The attenuated weight loss in mAb 8AP-treated animals indicates preserved metabolic function and reduced disease severity.

Collectively, these clinical parameters demonstrate that our model successfully replicates the systemic physiological derangements observed in human VREF sepsis and that mAb 8AP treatment provides measurable clinical benefit. Importantly, the improvement in clinical parameters persisted despite the continued presence of a bacterial burden at 96 h, suggesting that mAb-mediated immune modulation may confer clinical benefit through mechanisms beyond direct bacterial clearance, such as reducing the production of inflammatory mediators or enhancing immune cell function. This finding has important implications for the development of adjunctive immunotherapies for VREF infections, in which complete bacterial eradication may be challenging, whereas clinical stabilization can improve survival outcomes. In clinical practice, many septic patients survive despite persistent bacteremia when the inflammatory response is controlled and organ function is preserved; our data suggest that mAb therapy may help achieve this goal.

### 4.5 Immunological validation through cytokine profiling

The cytokine profiling data provide molecular-level validation of the systemic inflammatory response in our model and offer mechanistic insights into both disease pathogenesis and therapeutic mechanisms. The cytokine signature observed—particularly the elevation of IL-6, IL-8, and IL-10—closely parallels that reported in human VREF sepsis patients (Neves et al., 2015; Ameen et al., 2025; Vella et al., 2025), confirming that our rabbit model replicates not only the clinical manifestations but also the underlying immunological mechanisms of the human disease. IL-6, a pleiotropic pro-inflammatory cytokine and an established biomarker of sepsis severity in humans (Ameen et al., 2025; Vella et al., 2025), was significantly elevated in VREF-infected rabbits. IL-6 is produced primarily by monocytes/macrophages and endothelial cells in response to bacterial pathogen-associated molecular patterns (PAMPs) and drives the acute-phase response (Vella et al., 2025), including fever, hepatic acute-phase protein synthesis, and leukocyte mobilization. Clinically, IL-6 levels are associated with sepsis severity, organ dysfunction scores (SOFA, APACHE II), and mortality risk. The IL-6 levels observed in our infected rabbits were comparable to those reported in human VREF bacteremia patients (Ameen et al., 2025), supporting the translational relevance of this model. IL-8 (CXCL8), a potent neutrophil chemoattractant (Steven L. Kunkel, 1990), was markedly elevated, reflecting robust neutrophil recruitment to sites of infection. This finding aligns with our histopathological observations of extensive neutrophilic infiltration in affected organs, particularly the liver. IL-8 is a key mediator of the innate immune response to bacterial infection and is essential for neutrophil-mediated bacterial clearance (Vella et al., 2025). However, excessive IL-8 production can contribute to tissue damage via neutrophil-mediated proteases and the release of reactive oxygen species. IL-10, an anti-inflammatory cytokine produced by monocytes, macrophages, and regulatory T cells, was concurrently elevated, suggesting activation of compensatory anti-inflammatory mechanisms (Neves et al., 2015; Vella et al., 2025). IL-10 limits excessive inflammation by inhibiting the production of pro-inflammatory cytokines and antigen presentation. The IL-10/IL-6 ratio in infected animals (0.58–0.79) indicates a predominantly pro-inflammatory state, consistent with the acute phase of sepsis. In human sepsis, an early pro-inflammatory phase (characterized by high IL-6, TNF-α, and IL-1β) is often followed by a compensatory anti-inflammatory response (characterized by high levels of IL-10 and IL-4), and the balance between these phases influences outcomes. Our data suggest that at 96h post-infection, the rabbits remained in a predominantly pro-inflammatory state.

The observed cytokine profile characterized by elevated IL-6, IL-8, IL-10, IL-1β, and other mediators closely mirrors the cytokine signature reported in patients with human VREF bacteremia (Londoño et al., 2008; Al-Qahtani et al., 2024; Vella et al., 2025), further validating the translational relevance of this model. This concordance is particularly important for evaluating immunomodulatory therapeutics, as the rabbit immune system’s response to VREF infection appears to more faithfully recapitulate human cytokine cascades than rodent models, which often exhibit divergent cytokine profiles due to differences in TLR signaling and immune cell populations.

The trend toward lower pro-inflammatory cytokine levels in mAb 8AP-treated animals suggests that antibody therapy works through multiple mechanisms: (1) direct bacterial opsonization and clearance, reducing PAMP exposure and subsequent TLR activation (Huebner et al., 2000; Rossmann et al., 2015); (2) reduction of inflammatory mediators through decreased bacterial load; and (3) direct immunomodulatory effects through Fc receptor engagement on immune cells, which can influence the production of cytokines (Neves et al., 2015; Al-Qahtani et al., 2024; Vella et al., 2025).

The observed variation in cytokine responses among individual animals mirrors that seen in human patients and underscores the importance of personalized medicine approaches in the management of sepsis. This variability may reflect differences in individual immune competence, baseline inflammatory status, or genetic factors influencing cytokine production. Understanding the sources of this variation is important for identifying patients who are most likely to benefit from specific therapeutic interventions.

Future studies should include serial cytokine measurements at multiple time points (e.g., 0h, 12h, 24h, 48h, 72h, and 96h) to characterize the temporal dynamics of the immune response and correlate cytokine kinetics with clinical outcomes and bacterial clearance. Such data would enable identification of prognostic biomarkers (e.g., early IL-6 levels predicting mortality risk (Ameen et al., 2025; Vella et al., 2025)) and therapeutic windows for intervention (e.g., optimal timing for immunomodulatory therapy based on the cytokine phase). Additionally, measuring mediators such as procalcitonin, C-reactive protein, and damage-associated molecular patterns (DAMPs) would provide a more comprehensive picture of the host response and could identify additional therapeutic targets.

### 4.6 The therapeutic potential of monoclonal antibody 8AP

Rabbits treated with mAb 8AP showed significant improvements in clinical parameters (heart rate, respiratory rate, and body weight) compared to the untreated infected rabbits, despite a persistent bacterial burden at 96h. This finding suggests that mAb-mediated immune modulation may confer clinical benefits through mechanisms beyond direct bacterial clearance, including reductions in inflammatory mediators and enhancements of immune cell function. An important limitation of the current study is the absence of an isotype-matched non-specific control antibody. While our results demonstrated significant therapeutic benefits of mAb 8AP treatment compared to the saline controls, we cannot definitively exclude the possibility that some effects may be attributable to non-specific Fc-mediated immune modulation rather than antigen-specific opsonization alone. The observed improvements in clinical parameters could theoretically have resulted from: (1) specific opsonization and enhanced bacterial clearance by mAb 8AP, (2) non-specific immune modulation by any IgG antibody, or (3) Fc-mediated effects independent of antigen specificity.

However, several lines of evidence support the specificity of mAb 8AP effects. First, mAb 8AP demonstrated significant bacterial killing in our *in-vitro* opsonophagocytic assays (Section 3.3, **Figure 3**), indicating specific opsonization capability. Second, mAb 8AP specifically recognized polysaccharide structures on the VREF E155 cell wall, confirming antigen-specific binding. Third, the therapeutic effects were correlated with the presence of this specific mAb, rather than with any protein or antibody. Nevertheless, we acknowledge that future studies should include an isotype-matched non-specific control antibody (e.g., an IgG1 antibody against an irrelevant antigen, such as hen egg lysozyme) to establish the specificity of the therapeutic effects. This will be a priority in our ongoing research and is essential for advancing this therapeutic approach toward clinical translation.

### 4.7 Comparative advantages of the rabbit model over murine models

While murine models have been extensively used to study enterococcal infections, including several published VREF bacteremia models (Kak et al., 2000; Bosze and H. L. M., 2006; Kathleen Pritchett-Corning et al., 2011; Peng and K. J. H. K., 2015; Chambers et al., 2016; Esteves et al., 2018; Castañeda et al., 2021; Soares et al., 2022; Patricia V. Turner, 2025), our rabbit model offers distinct translational advantages that justify its development and adoption. First, rabbits have significantly greater phylogenetic proximity to humans than rodents, with less genetic divergence between rabbits and humans than between mice and humans (Soares et al., 2022). This is particularly relevant for immune system studies, as rabbits possess Toll-like receptor (TLR) signaling pathways that more closely mirror the human TLR distribution and function than those of mice (Vaure and Liu, 2014; Goh et al., 2017; Duan et al., 2022; Soares et al., 2022), which show marked divergence in TLR-mediated pathogen recognition. For example, human TLR10 is functional but absent in mice, while mouse TLR11–13 are absent in humans (Duan et al., 2022). This divergence can lead to misrepresentation of host-pathogen interactions and of immune cascade activation, a critical factor in human VREF bacteremia.

Second, the intermediate body size of rabbits (2–4 kg *vs*. 20–30 g for mice) provides substantial practical advantages for serial sampling and monitoring (Bosze and H. L. M., 2006; Peng and K. J. H. K., 2015; Goh et al., 2017; Esteves et al., 2018; Patricia V. Turner, 2025). Rabbit models allow for repeated blood sampling (0.5–1 ml per time point) without compromising animal welfare or significantly affecting circulating blood volume (total ~200–300 ml in rabbits *vs*. ~2 ml in mice), enabling detailed pharmacokinetic and pharmacodynamic studies that are challenging in mice. The larger organ size also facilitates more comprehensive histopathological analysis, enabling multiple analytical techniques to be employed on the same organ (e.g., bacterial load quantification, histology, immunohistochemistry, and molecular analysis across different sections), thereby reducing inter-animal variability and improving data quality.

Third, existing mouse models of VREF infection have primarily focused on peritonitis, urinary tract infections, or device-associated infections^10^. In contrast, our rabbit model specifically addresses acute bacteremia and sepsis independent of medical interventions. This distinction is clinically important, as primary VREF bacteremia (not associated with catheters or implants) represents a significant proportion of clinical cases and has a distinct pathophysiology. Mouse bacteremia models often require immunosuppression (e.g., cyclophosphamide), potentially altering host-pathogen dynamics and limiting translational relevance.

Fourth, the rabbit cardiovascular system more closely resembles the human system in terms of heart rate, blood pressure dynamics, and cardiac output relative to body weight, making rabbits superior for studying sepsis-associated cardiovascular dysfunction (Bosze and H. L. M., 2006; Kathleen Pritchett-Corning et al., 2011; Peng and K. J. H. K., 2015; Esteves et al., 2018; Patricia V. Turner, 2025). The clinical parameters we monitored (heart rate, respiratory rate, temperature) are directly comparable to the human SIRS criteria, facilitating the translation of findings to clinical settings.

However, we acknowledge that mouse models retain certain advantages, including lower costs (housing and purchase price), shorter generation times, established genetic manipulation tools (knockout and transgenic strains for mechanistic studies), and extensive availability of species-specific immunological reagents (antibodies, cytokine assays, and flow cytometry panels). The choice between rabbit and mouse models should be guided by the specific research question: mouse models are well suited for genetic mechanistic studies, high-throughput screening of compounds, and investigations of specific immune cell populations using genetically modified strains, while rabbit models offer superior translational relevance for studies aimed at clinical application, particularly for evaluating immunotherapeutics, understanding human-like immune responses, and assessing interventions that require serial sampling or detailed physiological monitoring.

In summary, our rabbit model complements rather than replaces existing mouse models, providing a valuable intermediate step between rodent studies and human clinical trials. The rabbit model is particularly suited to preclinical evaluation of novel therapeutics, including monoclonal antibodies, vaccines, and immunomodulatory agents, in which human-like immune responses and the ability to perform detailed physiological monitoring are critical for translational success. We envision a tiered approach to VREF research: initial mechanistic studies and high-throughput screening in mice, followed by translational validation in rabbits before advancement to human clinical trials.

### 4.8 Clinical and translational implications

This study provides a validated platform for evaluating novel therapeutics for VREF infections. The model’s ability to replicate human disease features, including SIRS criteria fulfillment, organ-specific pathology, and cytokine profiles, makes it particularly valuable for preclinical testing of antibiotics, immunotherapy, and combination treatments. The demonstration that mAb 8AP treatment improved clinical outcomes despite a persistent bacterial burden suggests that immunotherapy may be an important adjunct to conventional antibiotics, particularly in cases where bacterial eradication is challenging.

The finding that hepatic pathology was most severe despite a lower bacterial burden underscores the role of immune-mediated tissue damage in VREF sepsis and suggests that hepatoprotective strategies may be warranted in addition to antimicrobial therapy. Future therapeutic development should consider interventions that balance bacterial clearance with modulation of excessive inflammatory responses to minimize collateral tissue damage.

### 4.9 Limitations and future directions

Several limitations of this study should be acknowledged. First, the absence of an isotype-matched control antibody limits our ability to definitively attribute therapeutic effects to antigen-specific mechanisms. Second, the sample size was relatively small (*n* = 5 per group), limiting statistical power for detecting some differences, particularly in mortality. Third, the study evaluated only a single time point (96h) for cytokine analysis and organ pathology; serial sampling would provide a more comprehensive understanding of disease progression and therapeutic effects. Fourth, we did not assess the bacterial load in blood at 96h or measure mAb levels in plasma to correlate with clinical outcomes. Fifth, the model used immunocompetent rabbits, whereas many clinical VREF infections occur in immunocompromised patients; future studies should evaluate the model in neutropenic or immunosuppressed rabbits to better simulate high-risk patient populations.

Future studies should address these limitations and expand the model’s utility by (1) incorporating isotype-matched control antibodies, (2) increasing sample sizes for adequately powered efficacy studies, (3) performing serial sampling for temporal analysis of disease progression and immune responses, (4) evaluating combination therapies (antibiotics plus immunotherapy), (5) testing the model in immunocompromised rabbits, (6) assessing different routes and dosing regimens for mAb administration, and (7) evaluating additional therapeutic candidates, including vaccines and small-molecule immunomodulators.

## 5 Conclusions

This study successfully established a novel rabbit model of acute VREF bacteremia that accurately replicated key clinical, pathological, and immunological features of human infection. The model demonstrated (1) consistent induction of sustained bacteremia and sepsis, (2) systemic bacterial dissemination with organ-specific pathology, (3) fulfillment of SIRS criteria, (4) cytokine profiles comparable to human VREF sepsis, and (5) clinically relevant mortality. Treatment with the monoclonal antibody 8AP significantly improved clinical parameters (heart rate, respiratory rate, and body weight) despite a persistent bacterial burden, suggesting that immunotherapy exerts benefit through multiple mechanisms, including direct bacterial opsonization and immune modulation.

This rabbit model provides a valuable translational platform for evaluating novel prophylactic and therapeutic interventions for VREF infections, including antibiotics, immunotherapies, and combination treatments. The model’s ability to bridge the gap between rodent studies and human clinical trials makes it particularly suited for preclinical development of urgently needed therapies for this high-priority WHO pathogen. Future studies should focus on optimizing immunotherapeutic approaches, including the use of isotype-matched controls, evaluating combination therapies, and assessing the model in immunocompromised hosts to better reflect high-risk patient populations.

## Data Availability

The raw data supporting the conclusions of this article will be made available by the authors, without undue reservation.

## Supplementary material

Supplementary Table 1 Clinical characteristics among infected versus control groups. Data are presented as Mean ± SD. Statistical comparisons were performed using Fisher’s exact test for categorical variables.
