# Supplementary material for: A rabbit model of acute bacteremia and sepsis caused by vancomycin-resistant Enterococcus faecium
Source: Front Cell Infect Microbiol. 2026 Apr 16;16:1619290. doi: 10.3389/fcimb.2026.1619290 (PMC13128805; doi:10.3389/fcimb.2026.1619290)
Supplement: Supplementary Table 1 — Clinical characteristics among infected versus control groups. Data are presented as Mean ± SD. Statistical comparisons were performed using Fisher’s exact test for categorical variables. [file Table1.docx]

**Supplementary Table 1. Clinical Characteristics among Infected vs Control Groups**

| Characteristic | VREF-Infected  (Groups 1+2, n=10) | Saline Control  (Group 3, n=5) | p-value |
| --- | --- | --- | --- |
| Weight loss >10% | 80.0 ± 12.6% | 0.0 ± 0.0% | <0.001 |
| Fever (>40°C) | 100.0 ± 0.0% | 0.0 ± 0.0% | <0.001 |
| Lethargy (score ≥2) | 70.0 ± 14.5% | 0.0 ± 0.0% | <0.001 |
| Positive blood culture | 100.0 ± 0.0% | 0.0 ± 0.0% | <0.001 |
| Mortality (96h) | 20.0 ± 12.6% | 0.0 ± 0.0% | 0.089 |

*Data are presented as Mean ± SD. Statistical comparisons were performed using Fisher’s exact test for categorical variables*[*https://www.frontiersin.org/articles/10.3389/fcimb.2026.1619290/ full - supplementary-material*](https://www.frontiersin.org/articles/10.3389/fcimb.2026.1619290/%20full#supplementary-material)*.*
